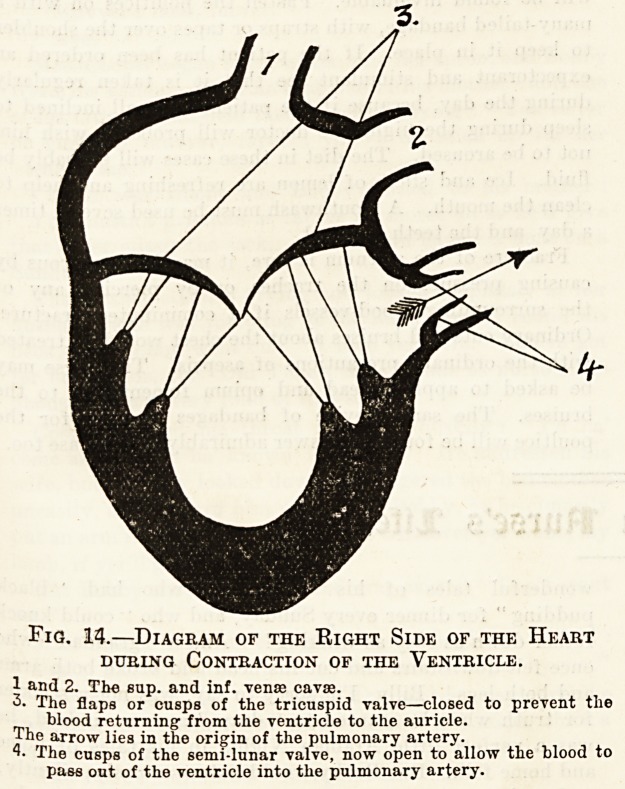# "The Hospital" Nursing Section

**Published:** 1906-04-21

**Authors:** 


### The Hospital.

### Nursing Section.

Contributions for "THE HOSPITAL," should be addressed to the EDITOR, "THE HOSPITAL" NURSING SECTION, 28 & 29 Southampton Street, Strand, London, W.C.

No. 1,021.—VOL. XL. SATURDAY, APRIL 21, 1906.

### Notes on News from the Nursing World.

### RESIGNATION OF THE MATRON OF GRIMSBY HOSPITAL.

WE regret to announce the resignation of Miss Frances Crichton from the post of matron of Grimsby and District Hospital. This step has been taken by Miss Crichton solely on account of her health, and it was with much reluctance that she felt compelled to decide to retire from hospital work for a time, at any rate. At a meeting of the hospital Management Committee, the Chairman, Mr. C. F. Carter, read a letter from her in which, while intimating her resignation, she gratefully acknowledged the great help and encouragement she had always received from the Committee in her work. He then moved a resolution expressing regret at Miss Crichton's resignation and high appreciation of her services. In the course of his speech, Mr. Carter said that if it had not been for the matron's splendid administrative ability it would have been impossible for the managers to have carried out the large operations they had initiated during her tenure of office; and Dr. Westlake, on behalf of the honorary medical staff, joined in paying a tribute to her abilities. We hope that Miss Crichton, whose work at Grimsby was described in our issue of October 14, 1905, soon after the opening of the addition to the Nurses' Home at Grimsby, will soon find herself strong enough to resume her career.

### SCARCITY OF SISTERS.

WE learn, with reference to a vacancy for a sister at the Royal Albert Edward Infirmary and Dispensary, Wigan, that no appointment will be made for the present owing to the fact that no really well-qualified candidate has applied for the post. This is rather surprising, and cannot be due to any doubts about the standing of the institution. The hospital, which was opened in 1873, has a resident medical staff, and contains 148 beds. Probationers are trained for three years, and the nursing staff consists of a matron, assistant matron, six sisters, twelve nurses, and fourteen assistant nurses and probationers.

### NURSING INSTITUTIONS AND REGISTRATION.

THE subscribers to the Leicester Institution of Trained Nurses were invited at the annual meeting to pass a resolution in favour of State Registration. Dr. Pope, the author of the proposal, remarked that it had nothing to do with the Institution, an observation in which we entirely concur. That being so, it seems a pity that he should have introduced the subject, especially as he proceeded to urge that the Government should be importuned to do its utmost "to carry the Bill." It is true that the resolution was carried and so far Dr. Pope gained his point; but, in the present condition of the controversy, when the opinion on Registration is not only hopelessly divided, but the advocates of it are—as Miss Forrest showed in her letter to us last week—at loggerheads among themselves, it cannot be to the advantage of nursing institutions partly dependent upon charity that motions like that of Dr. Pope should be sprung upon meetings called for the ordinary purposes of business.

### MATERNITY NURSES AT A DISCOUNT.

QUESTIONS are often addressed to us by nurses who wish to add maternity training to their curriculum, or from correspondents who are unable for some reason to take a three years' course of general nursing. We always endeavour to convince them of the advisability of qualifying as a midwife rather than as a maternity nurse only, and a fact which has lately come to our knowledge has strengthened our belief that as the years go on the demand for this special class of nurse will diminish, and it will become increasingly difficult for them to obtain employment. A lady who was expecting her first baby, before engaging a nurse, spoke to her medical man on the subject, telling him that she had been recommended one who had received her three years' general training and obtained a certificate as a maternity nurse at an important London lying-in hospital. The medical man, although he had no reason to believe that it was likely to be a difficult case, at once said that unless the nurse held the certificate of the Central Midwives Board, he would prefer that she should not be engaged.

### WAITRESSES ATTIRED AS NURSES.

THERE are some abuses of the use of a nurse's uniform which it is not easy to deal with; but we think that in the case of the waitresses at an establishment in Bournemouth, to which reference has been made by some of our correspondents, an effective protest might possibly be made. The number of trained nurses in Bournemouth is considerable, and we suggest that they should send the proprietors of the tea rooms in question a courteous request asking them to adopt some other costume for their employés. It is quite possible that nurses' attire was chosen merely because it was thought becoming, and without any idea of the annoyance it would naturally give to those who alone have the right to wear the uniform.

### CHANGES AT THE NURSES' HOSTEL.

We understand that Miss C. J. Wood has ceased to act as superintendent of the Nurses' Hostel in Francis Street, and is succeeded by Miss A. E. Hulme. Miss Wood, however, retains the post of managing director, and there is, happily, no occasion to treat her career as closed. It is fortunate for the shareholders in the Nurses' Hostel, which she originated and founded, that she will continue to be closely associated with its management, and that in the person of Miss Hulme the institution will possess a superintendent of proved capacity, who, as superintendent for several years of the Nursing Sisters' Institution in Devonshire Square, gave evidence of the knowledge, administrative abilities, and tact which are essential to success in the new duties she has undertaken.

### GARMENTS AND LINEN.

In connection with the recent meeting of the Paddington Branch of St. Mary's Hospital Ladies' Association, we hear that in one ward of the hospital 935 garments of all kinds for the patients were on view. The matron and sisters, who were entertained by the members of the Association to tea, naturally expressed their gratification at the substantial result of their labours. We are glad to learn that an association on kindred lines has been established at Cardiff and promises to make excellent progress. A large committee has been formed, with an unlimited number of associates, each of whom is required to contribute not less than two linen articles, chosen from a long published list. These articles are all to be handed in on a given date, and Saturday, November 3, has been fixed as the day for presenting the entire collection to Cardiff General Infirmary. The movement has caught on to such an extent at Cardiff that already the house committees of the other hospitals and infirmaries in South Wales are preparing to form similar organisations. We are sure that their multiplication, and the consequent practical help thus given to the institutions, will be particularly appreciated by matrons. Our own columns constantly bear testimony to the need in respect to garments, and if the question of the renewal of the linen supply, which often causes much anxiety, can be solved by special efforts in some cases, we do not see why it should not in others.

### NURSES FOR NICE.

As we have received several inquiries from nurses as to the probability of openings for them at the new Queen Victoria Memorial Hospital at Nice, we think it well to state that at present the staff only consists of the matron, Miss Schappera, who was trained at the Alfred Hospital, Melbourne, and served throughout the South African War, and one permanent trained nurse. Others will be engaged in the neighbourhood as occasion requires. Miss Schappera has received numerous applications for the post, and will probably select from these for next year. The size of the nurses' sitting-room, which is unusually commodious for a small hospital, suggests that probably a larger staff will be required anon. The matron's and nurses' quarters are connected with the hospital by a covered passage, and the view from some of the rooms over the beautiful Baie der Anges must be a constant joy to the inmates.

### WEST OF ENGLAND GUARDIANS AND DISTRICT NURSING.

At a recent meeting of the Penzance Guardians it was proposed that the sum of two guineas per annum should be paid to each of the nursing associations in the districts. In advising this action, one of the Guardians mentioned that in the parish of St. Erth two years ago they were paying a woman considerably more than £5 a year to look after one patient. To illustrate another aspect of the case, he stated that in another parish, that of Ludgvan, a pauper patient had lately been attended by the district nurse for the last two months twice a day, and remarked that had the Guardians been called upon to pay for the services rendered, the cost would have been considerably more than they were asked to subscribe to the local association for the year. It can be judged from these examples that, in the interests of economy alone, the Penzance Guardians have arrived at a wise decision.

### SOUTH LONDON DISTRICT NURSING ASSOCIATION.

Princess Louise, Duchess of Argyll, has intimated her intention of being present at the annual general meeting of the South London District Nursing Association, which will be held at the Mansion House on Wednesday, May 9, at 3.30 p.m. The report which has just been issued shows that the number of patients attended was 2,176, and the number of visits paid 44,222. In addition to these, 163 visits were paid to persons whom it was not found necessary to attend a second time. Although the Committee have cleared off a deficit of £40 and increased the amount of subscriptions and donations by nearly £100, the yearly income does not yet suffice for the yearly expenditure, there being a difference of £57 on the wrong side. The staff in the home is unchanged, but two of the nurses were incapacitated by illness last year, and there were difficulties in carrying on the work with intermission; but they were overcome, and the whole of the nurses are now available. One of the features of the report is the table of specimen cases from the superintendent's register; it is very explicit, and furnishes quite an object lesson in district nursing.

### BOLINGBROKE HOSPITAL.

On Monday in Holy Week the Bishop of Kingston paid a visit to the Bolingbroke Hospital, Wandsworth Common, and conducted a special service held for the nursing staff, at which he delivered an impressive address. Next month the nurses of this hospital will have the pleasure of welcoming the Princess Royal, who has consented to receive purses containing donations to the fund now being raised to defray the cost of the first wing of the reconstructed building, when the foundation-stone is laid on May 5.

### LECTURES TO MIDWIVES.

The new syllabus, to cover a course of 20, instead of 13, lectures to midwives under the London County Council, has been issued. It closely resembles the old syllabus, except that such subjects as sepsis, abnormal labour, and hæmorrhage are more elaborately dealt with. Prominence is also given to the feeding of infants and infantile diseases. Only one lecture, as formerly, is given at the College of Surgeons for practical demonstration of anatomy; though, with the prolonged course, two might very profitably have been held.

### THE HOLIDAY QUESTION AT BRISTOL.

The Bristol Guardians, in deciding to act upon the recommendation of their Hospital Committee, who advised that three weeks' holiday should be allowed to the nurses in their employ, passed an amendment limiting the operation of the rule to the present year. The amendment received 31 votes, as against 17 in favour of making the rule permanent. We doubt, however, whether, the concession having once been made, the Bristol Guardians will be able to shorten the vacation, even if they wish to do so.

### DISTRICT NURSING IN CORNWALL.

At the annual meeting of the Cornwall County Nursing Association, Lord Mount-Edgcumbe in the chair, it was stated that during the year three entirely new district nursing associations had been formed, for St. Paul and Mousehole, Saltash, and St. Buryan. The number of affiliated district nursing associations is 37. The number of nurses employed is 42. Seven probationers are now undergoing training, six at Padstow, and one at Truro. When these have completed their training the Association will have trained 53 nurses. The Association's income for the year was £620 17s. 6d., and of the expenditure of £668 19s. 5½d., no less than £414 5s. 10d. was directly contributed to the district nursing associations in the shape of training village nurses, who on the completion of their training worked for the district nursing associations at low salaries in order partly to repay the Association outlay, and in the shape of bonuses to nurses who had completed the three years' service for which they bound themselves in return for the training. The District Associations spent £2,692, as compared with £2,928 spent last year. During the year the County Superintendent paid 180 visits of inspection to nurses, attended five meetings to afford information as to starting districts and nineteen District Nursing Committee meetings, had 177 interviews on matters connected with the Association, wrote 1,858 letters, and spent 16 days nursing in districts requiring nursing help. The Chairman, in moving the adoption of the report, referred to the fact that the population cared for by the Association was 139,000. Lady St. Germans and Lady Margaret Boscawen were asked to represent the Association at the meetings in London of the Queen's Institute and of the representatives of County Nursing Associations.

### A QUESTION OF CAPACITY.

The Coroner for Barnstaple held an inquest this week on the body of a labourer's wife who on Monday gave birth to twins. She was attended by a certificated nurse, who, observing, an hour after the birth, that the patient was not making the progress she should, sent for a doctor, but death took place from hæmorrhage before his arrival. The doctor, who got to the house 20 minutes after he had received the message, stated in evidence that if a medical man had been called in in the first instance her life would have been saved; and the Coroner then expressed the opinion that "the nurse was really too old for the work." In this opinion the jury concurred, and added to the verdict of death from natural causes a rider that they did not think it advisable that she should attend any more cases unless a doctor was also in attendance. The nurse is sixty-six, and though it is of course quite possible for a nurse of that age to be quite fit for her work, we should say that the exception proves the rule.

### QUEEN'S NURSES AND SMALL FEES.

At the nineteenth annual meeting of the Hereford Nursing Association—the first held since its affiliation to the Queen Victoria Jubilee Institute—the Mayor, who presided, said that it had occurred to him whether the committee could provide an extra nurse for attendance on patients who could afford to pay sixpence or a shilling a visit. A member of the committee rejoined that the matter had already been considered, but that they had not been able to formulate any scheme by which it could be carried out. Another speaker deprecated any paying scheme in connection with a charity, and this objection is, as a matter of fact, insuperable to any organisation employing Queen's Nurses. But if, as stated, there is a great need in Hereford for a nurse to attend cases in families of persons of small means, it seems a pity that a plan cannot be devised by local ingenuity to supply it.

### A HEAVY DEBT AT CARDIFF.

A striking feature of the report of the Cardiff and District Branch of Queen Victoria's Jubilee Institute, which was read at the annual meeting, is that the nurses paid 4,000 visits more in 1905 than in the previous year. While this, of course, shows that there is great scope for the Queen's nurses in Cardiff, we regret that the speakers had to deplore a debt of £631. It is fair to say that the debt is probably largely due to the expenses incurred in starting the Maternity Home, but we are not sure that this risk should have been run. It would have been more satisfactory to have obtained the whole of the special sum required before the department was decided upon, and certainly the first effort of the fifteen ladies and gentlemen who have just been apointed as the Committee should be to put the organisation upon a sound financial basis by means of an adequate number of regular subscribers who, in such an exceedingly prosperous city as the metropolis of South Wales, should be quite easy to obtain.

### The Nursing Outlook.

"From magnanimity, all fears above;
From nobler recompense, above applause,
Which owes to man's short outlook all its charm."

### NURSING IN IRELAND.

A Hospital Physician has written a long article on hospital nurses, their Ameliorations and Prospects, in the Dublin *Evening Telegraph*. He specially condemns the system prevailing in district or union hospitals, which, with the exception of those in the north of Ireland, employ nurses at from £35 to £50 per annum all found, who have to remain nearly always on night duty. "Hospital Physician" maintains that the matrons and nurses of these union hospitals are generally members of some religious order, who are very willing to take up these positions, but not so willing to discharge their full obligations. The matrons and nurses referred to, for instance, avoid doing night duty, and thus put the Guardians to the expense of providing skilled nurses for this work. He maintains that the result of this absence of a sense of duty on the part of those in authority is that the skilled night nurses are constantly changing, and very seldom keep an appointment for longer than one year. He gives instances of union hospitals where constant resignations have taken place, and mentions one where the Guardians fail to get trained nurses for night duty. We have no personal knowledge of the facts, but if they are as stated we agree with "Hospital Physician" that where nuns are employed to act as the nursing staff of union hospitals they must be trained nurses, and must be prepared to discharge the usual and full obligations of an ordinary nurse in a large hospital. It appears that some religious orders, the Bon Secours, for instance, are fully alive to their responsibilities, and do in fact take their turn at night and day duty as they did when they were probationers.

Having stated the objections to the present want of system in district and union hospitals in Ireland, as set forth by "Hospital Physician," it may be interesting to indicate the remedies he suggests. He demands "a better and kindlier care and supervision of the probationers, especially on the part of the matrons," who, he regrets to say, "are answerable for a good deal of the neglect and severity towards these young girls." He insists that during their probation no nurse should be allowed out after dark. During the winter months, from October to the end of March, off-duty hours should be from two to five o'clock each day; during the summer months, from April to the end of September, the hours of recreation should be from five to eight o'clock. He strongly advocates that outdoor uniform should always be worn. "Hospital Physician" considers that no nurse should be on night duty for longer than six hours. To effect this he recommends that each nurse should go on duty at 8 P.M., and be relieved at 2 A.M. by a nurse who will remain in the ward to 8.30 A.M., when she hands over her charge to the day nurse. He also urges that no nurse should get up in the morning till 7 A.M., and maintains that these changes from early morning rising and night duty are absolutely necessary for the preservation of the health of the nurses. He points out that in no other department of life is a longer night duty than four hours permitted, and instances the Army, Navy, police, and coastguard services. Young women probationers are not so strong as the men in these services, nor are their duties less onerous. He adds, however, that when nurses are employed on private cases their night duty might be extended to eight hours, and concludes by affirming that he has expressed the considered opinion of most of the medical and surgical staffs of Irish hospitals.

We shall be glad to have the views of our Irish readers upon the points raised by a Hospital Physician. Several earnest and excellent people have endeavoured to secure an eight hours' day for the working nurse without success, and though we are of opinion that it is desirable to reduce the working hours as much as possible, we regret that the difficulties which have to be overcome have so far proved insurmountable. The best Roman Catholic opinion in this country now insists that all nuns who act as attendants on the sick shall first undergo an adequate course of hospital training. We are confident that no member of a religious order who is also a qualified nurse would desire to shirk her duty in any way; and we trust that "Hospital Physician" may be labouring under some misapprehension when he states that the matrons and nurses, being members of religious orders, who are working in district or union hospitals in Ireland, fail to discharge their obligations and shirk night duty systematically. In many Irish hospitals the nursing and system of training leaves little or nothing to desire. We hope, therefore, that a free discussion of the position of Irish nursing as it is to-day may secure adequate reform in district, union, and other hospitals which are at present not so efficient in nursing matters as they undoubtedly ought to be. The best nursing opinion in Ireland is as desirous to secure the highest efficiency as that of any country in the world. If there were more voluntary effort and less local government in hospital and nursing affairs, important and necessary changes would promptly be introduced. The strongest argument against State supported and managed hospitals is the extravagance and monotonous inefficiency which too often results. We shall be pleased if our Irish readers will express their views on "Hospital Physician's" criticisms in The Hospital.

### The Care and Nursing of the Insane.

By Percy J. Baily, M.B., C.M.Edin., Medical Superintendent of Hanwell Asylum.

### I.—ANATOMY AND PHYSIOLOGY.

(*Continued from page 7.*)

### The Blood Circuit.

We must now trace the course which the blood follows during its circulation or journey from the heart, through the arteries, capillaries, and veins, back again to the heart (fig. 12). We shall find that during a complete circuit the blood passes

Fig. 12.—Diagram of the Blood Circuit.

1. Capillaries of Head and Arms. 2. Pulmonary Capillaries (Lungs). 3. Capillaries of Stomach and Intestines. 4. Capillaries of Body. 5. Superior Vena Cava: Brings blood from head and arms. 6. Branch of Aorta. 7. Aorta. 8. Portal Vein. 9. Inferior Vena Cava: Brings blood from all parts of the body except head and arms. 10. Pulmonary Veins: Two from each lung. 11. Pulmonary Artery: Carries blood from right ventricle to lungs. * From the right ventricle through the lungs to the left auricle is the Lesser or Pulmonic Circulation. † From the left ventricle through the general capillaries of the body to the right auricle is the Greater or Systemic Circulation.

through the heart twice—once on the right side of that organ and once on the left. In tracing this journey it does not matter at what part of the circulation we begin, but it will be most convenient to start with the venous blood which is coming from the various systemic capillaries towards the right side of the heart. This blood, as we know, is gathered up by the veins and it is finally collected into two large venous channels. One of these is called the superior, the other the inferior vena cava.* The former contains the blood which has passed through the capillaries of the head, neck, and upper limb, the latter, that from the remaining parts of the body. These two veins pour their blood into the right auricle. When this chamber is filled with blood its walls contract and its contents (venous blood) are driven through the tricuspid opening into the right ventricle (fig. 13). As soon as this chamber is full its walls begin to contract and the flaps of the tricuspid valve are forced together by the pressure of the blood in the contracting ventricle, thus closing the orifice between the auricle and ventricle (fig. 14). Since the blood is thus prevented from returning to the auricle, its only means of escape from the ventricle is through the pulmonary artery which carries it to the lungs. After passing through the pulmonary capillaries it is brought back to the left side of the heart

Fig. 13.—Diagram of the Right Side of the Heart during Contraction of the Auricle.

The arrow lies in the auriculo-ventricular opening.
1 and 2. The superior and inf. venæ cavæ. The openings of these vessels into the R. auricle, as of those of the pulmonary veins into the L. auricle, are not guarded by valves, but are closed during contraction of the auricle by muscular action, only partially shown in the diagram.
3. Flaps or cusps of the tricuspid valve—open to allow the blood to pass from the auricle into the ventricle.
4. The origin of the pulmonary artery showing the semi-lunar valve closed to prevent the blood from returning to the ventricle while that chamber is being filled from the auricle.

by the pulmonary veins. Of these there are four, two from each lung. They pour their blood into the left auricle. On the left side of the heart the same sequence of events occurs as we have already seen on the right side, that is to say, as soon as the left auricle is full its walls contract and drive the blood (arterial) through the mitral opening into the left ventricle, and when this chamber is full its walls contract and, as in the case of the tricuspid opening, the mitral orifice is closed by the flaps of the mitral valve. The blood is then forced (under considerable

* The plural of these words is venæ cavæ.

pressure) into the main artery of the body, which is called the aorta, and is distributed by this vessel and its branches to the capillaries all over the body. As soon as the contraction of the ventricle is over, its walls begin to relax again, and therefore there is a tendency for the blood in the aorta (or pulmonary artery) to be forced back into the ventricle. The returning blood, however, distends the three pouch-like (semi-lunar or half-moon) valves, which, as we

Fig. 14.—Diagram of the Right Side of the Heart during Contraction of the Ventricle.

1 and 2. The sup. and inf. venæ cavæ.
3. The flaps or cusps of the tricuspid valve—closed to prevent the blood returning from the ventricle to the auricle.
The arrow lies in the origin of the pulmonary artery.
4. The cusps of the semi-lunar valve, now open to allow the blood to pass out of the ventricle into the pulmonary artery.

have seen, surround the origin of the aorta and pulmonary artery in the ventricle, causing their edges to meet in such a manner as to completely close up the orifice and thus prevent the blood from finding its way back to the ventricle.

The right side of the heart deals only with venous blood, whereas the left side is concerned only with arterial blood. All the arteries in the body convey arterial blood from the heart except the pulmonary artery which, as we have seen, carries the venous blood from the heart to the lungs. So with regard to the veins—all these vessels carry venous blood except the pulmonary veins which bring arterial blood from the lungs to the heart.

That portion of the blood-current which extends from the right ventricle through the lungs to the left auricle is called the pulmonary circulation; in it, as we have just seen, arterial blood is contained within the veins and venous blood within the arteries. That portion which extends from the left ventricle, through the vessels of the tissue generally back to the right auricle, is known as the systemic circulation, in it the arterial blood is contained within the arteries while the venous blood flows in the veins.

*The Portal Circulation.*—There is one vein in the body which differs from all the others in that it ends after the manner of an artery by dividing up into a series of small branches and capillaries. This vein is called the portal vein. It collects the blood from the walls of the stomach and intestines as well as from the pancreas and spleen, that is to say, the blood which contains the newly absorbed nourishment. It passes to the liver and divides up within that gland into a series of capillaries from which the blood is again collected by another vein (the hepatic vein) through which it passes into the inferior vena cava. The portal circulation is merely a part of the systemic circulation and its purpose is that the nourishment coming from the alimentary canal may be brought into contact with the cells of the liver before it is thrown into the blood-stream. Another peculiarity about this portal vein is that it contains no valves, in which it resembles the pulmonary and cerebral veins.

### The Nurses' Clinic.

### INJURIES OF THE CHEST.

Injuries to the chest if at all severe are always serious, complications frequently develop which give rise to the gravest symptoms.

The thoracic viscera—*i.e.* the lungs, the heart, and large blood-vessels—may be injured with fatal results, or the injury may be of a simple character, such as contusions or fracture of the ribs without internal injury.

From the nurse's point of view little perhaps can be done for the most severe chest injuries; they are so frequently fatal that little remains to be done but to make the patient as comfortable as possible by putting him in the most comfortable position, which is generally that of being propped up with pillows, and to administer warmth by means of hot blankets and bottles.

These injuries may be (*a*) laceration of the lungs with profuse hæmorrhage, (*b*) rupture of blood-vessels, (*c*) injury to the heart. Any of these will naturally nearly always prove fatal.

Other injuries may be (*a*) fracture of the ribs, (*b*) fracture of the clavicle, (*b*) fracture of the sternum, (*c*) separation of the costal cartilages. Fracture of the ribs may be caused by direct violence, such as a blow or kick, when the ribs will be fractured at the point of contact, or by pressure such as compression in a crowd or by machinery accidents, when the ribs will fracture at their angles or most convex point. When fractured by direct violence the sharp ends may tear the pleura and penetrate the lungs or heart. The complications to injuries of the lungs will probably be pleurisy, pneumonia, hæmoptysis or emphysema, which latter is caused by air escaping into the cells of the subcutaneous tissue.

Supposing the patient to be suffering from fractured ribs which have not penetrated the lung, the treatment is comparatively simple, but all the same he must be carefully and observantly nursed so as to give him as little pain and discomfort as possible in the first place, and in the second place to be able to report any fresh symptoms which may indicate the development of complications.

The doctor will probably have strapped or bandaged the

**THE NURSES' CLINIC—*Continued.***

chest so as to give some support and thus ease the pain caused by movement of the ribs during respiration and also to favour union by keeping the fractured ends in apposition.

The patient must not be allowed to move the trunk more than is necessary, he must be prevented from slipping down in the bed by a bed-rest—if the back of the bedstead does not provide this luxury—and plenty of pillows, also a firm pillow may be placed under the knees and another at the feet. If, after all this, he still slips, try an invention under the mattress made by two flat pieces of board, the one fastened in the centre at right angles, the other forming the base. This makes a firm ridge against which the patient sits. It is not as uncomfortable as it may sound, and it is a great relief to the sufferer not to have the pain of being hauled up in bed every hour or so.

The diet should be light and easily digestible for a few days, such as fish, milk puddings, eggs, bread and butter. These foods also have the advantage of being able to be eaten with a fork and spoon, so that there is not the exertion of cutting the food.

The temperature, pulse, and respirations must be watched, especially the latter, and reported if it quickens much in a short time; also the sputum should be watched for any signs of hæmorrhage from the lungs.

Keep the patient warm and free from draughts, and by the end of three or four weeks he will be quite convalescent, providing that no complication has hindered him.

If, however, he falls a victim to pleurisy or pneumonia he must be nursed with even more care. The same remarks about warmth and draughts will also apply here. Let him wear a flannel jacket opening down the back, so that there is no exertion when necessary to put on and remove. If the doctor orders poultices either of ice or linseed the split shirt will be found invaluable. Fasten the poultices on with a many-tailed bandage, with straps or tapes over the shoulder to keep it in place. If the patient has been ordered an expectorant and stimulant see that it is taken regularly during the day, because if the patient is at all inclined to sleep during the night the doctor will probably wish him not to be aroused. The diet in these cases will probably be fluid. Ice and slices of lemon are refreshing and help to clean the mouth. A mouthwash must be used several times a day, and the teeth cleansed.

Fracture of the sternum is rare, it may be dangerous by causing pressure on the trachea or by piercing any of the surrounding blood-vessels if a comminuted fracture. Ordinary cuts and bruises about the chest would be treated with the ordinary precautions of asepsis. The nurse may be asked to apply a lead and opium fomentation to the bruises. The same device of bandages as used for the poultice will be found to answer admirably in this case too.

### Incidents in a Nurse's Life.

### MY LITTLE HEART CASE

Bobby was a dear little heart case of six or seven, a most quaint little youth and adored by the whole ward almost as soon as he had been admitted.

Poor little chap, his was practically a hopeless case when he came in, and he used to almost climb up the side of his cot like a little bear, in his efforts to get breathing relief. His favourite sleeping posture (and sleep was a rare luxury with him) was kneeling against the side of his cot with his pillow bunched up and balanced on the top, and his head on his arms against it. He was a philosophical baby and never complained, taking his sufferings in a most seriously calm way. One morning, after an examination by the doctors, an odd whispered sentence or a look had evidently been noticed by Bobby—there was not much that escaped him—and I heard him tell his neighbour, a burn, that "doctor" thought him hopeless. He added: "I want ter die cos my bweaving's so bad an' it hurts me."

Billy called out "Aren't yer afraid, Bobby?" and he said, "Course I'm not, I'm a man, an Nurse Dowofy 'll come a bit of the way wiv me." Strange little ideas he had, and I was very, very fond of him, so I went to his bed and told him "I'll hold your hand and take you up to the gate, Sonny, but they won't let me in."

He put out a thin little arm and pulled my head down and whispered:

"When you come I'll ask them to let me open the door for yer, and I'll tell St. Peter how good ye were to little boys."

Dear little Bobby! perhaps my babies will some day, when I am in need of it, be allowed to put in a good word for me. We were very anxious when visiting day came round after his admittance to see what sort of parents he had, as "a neighbour" had brought him to "Out Patients," volubly explaining that his mother and father were away, but would be coming up shortly.

Bobby did not talk of "home" as the other children did, nor enlarge at all on his relations, in spite of Billy's wonderful tales of his "muvver" who had "black pudding" for dinner every Sunday, and who "could knock father down as easy as winking!" And a "grandad" who once fell downstairs and cut his head and broke both arms and both legs! Billy, I'm sorry to say, was not a stickler for truth when he wanted to "draw" anyone, indeed, he was a perfect little Arabian Nights in his tales of home and home folk. But Bobby would listen seriously, silently, and make no comment whatever. The first visiting day since his entrance came and went, but brought him no visitor. His was the one lonely cot unattended by the usual relative or friend on either side—for we allowed two to each child—but he gave no sign of loneliness and knelt up at the side regarding the visitors with solemn unblinking eyes.

Thinking he might be feeling his people's neglect of him acutely, I took some wool I was cutting to his bedside and told him I would be his visitor, and then, after a short silence, he informed me that his father did not live with his mother but "had another missis." These children are perfect little Methuselahs in their knowledge and ideas of sin in every form, but Bobby seemed to be very fond of his erring parent and expressed a wish to see him if I would write, and also asked me to send for his mother. He had evidently spent brief periods at both parents' houses and been intermittently looked after by the neighbour who had taken upon herself to bring him to us.

Two days after visiting day Bobby was taken very much worse, and it was easy to see that his sufferings would soon be done with. Having procured from him the addresses of his parents, I sent for them without delay, as the child seemed particularly anxious they should come together.

Mrs. Cooper turned up first, dirty and hatless, with straggling hair and pinned bodice, with a kind face and honest eyes, though, from what I gathered from Bobby, she, alas! was no better than her husband. She was crying bitterly and seemed devoted to Bobby and he to her, in spite of her never having been near hospital to see him.

The father ambled up ten minutes later, a gaunt, tall man, with an ashamed face and furtive eyes, and then a strange scene was enacted. There was the usual chair on either side of Bobby's cot, and Mrs. Cooper had taken one, so Mr. Cooper with a nod (!) to his wife, sat down in the other, possessing himself of Bobby's hand nearest him while Mrs. Cooper also held one.

"Hullo, farver," Bobby said, in his weak voice; he was sitting propped up, and was feeling better again.

"'Ullo!"

"Muvver's here, farver."

"Aye."

Mrs. Cooper began to cry again, into her apron, and Bobby freed his hands. Then he took one of his mother's and one of his father's, and held them together saying: "You've ter be good ter muvver, farver; you've ter stick up for her 'stead o' me."

Cooper was silent, and Bobby turned to his mother: "You mustn't grumble at farver no more; so as he'll give that other missis the sack. An' let him have his buns on a Sunday."

The mother stopped crying and looked helplessly at her husband, who was fumbling at the handkerchief round his neck, and Bobby went on: "I love yer bofe. You've got ter be friends, for 'Old Time's Sake,' same as nurse sings about" (he was very fond of that song).

Cooper cleared his throat and said huskily: "I'd ha' come afore if I'd ha' known 'bout him." He addressed his wife, but she only looked down and fingered the bed-clothes uneasily, and I heard him whisper to Bobby, as he clumsily put an arm round him, "Daddy'll do whatever yer want, my lamb, if yer'll get better."

But Bobby shook his fair head and panted "Don't want ter."

Mrs. Cooper burst out crying again, and her husband said: "Don't, Annie, don't; I'll come back and do right by yer."

"What about 'er?" I heard the mother say, and he answered in a shamefaced way: "She'll not mind, she's a regular bad 'un."

And Bobby murmured "A regular bad 'un."

I told them they must leave the child then for half an hour, and they both kissed him, and Cooper touched his wife's hand, which made her cry harder than ever. I saw them go down the street together, and when I told Bobbie so he gave a sigh of satisfaction and said: "It's 'Old Time's Sake' now, ain't it, nurse?"

Dear little chap! he had another attack, and when they returned he could not speak to them. He held out his arms to me and I leant his head against my shoulder. "He's fond of yer, nurse, bless yer!" sobbed his mother, and Cooper muttered: "She's been right good ter him, I doubt."

I motioned to him to take his wife's hand, and I put Bobbie's over them. He just smiled at his father and said: "I wanted yer farver—'Old Time's Sake'" and went very quietly without any pain.

Mrs. Cooper cried out: "Oh, 'e's gone, 'e's gone, my little lad!" and Cooper with a great sob went to her and put his arm round her shoulders.

Later on in the day, when I took the parents to our beautiful little mortuary, I had a talk with them, and Cooper, wringing my hand—he was crying great tears all the time—assured me that he and his wife should do what Bobby wanted and "be friends" again for always.

I see them now and again and they are very happy, poor, dirty, and slipshod though they be; but they always wish they had been together in Bobby's lifetime, poor things!

I gathered from Mrs. Cooper that she and her husband parted when Bobby was two years old, and the child had gone from one to the other ever since.

### Effect of Training on Character.

Someone said to me just now, "Nurses always seem to have so much more 'go' in them than ordinary women." The obvious answer was that if they had not they would be of little use to their patients.

Now where do they get their "go" from? When the aspirants make their first appearance in the matron's office, with anxious mien stammering out their lifelong (usually lifelong) desire for training, there is no visible difference between them and the first half-dozen young women you may meet any day in the street, whose desires do not to all appearance rise beyond the finery in the shop windows they are passing. But in three years' time those same young women will probably be found ruling a ward of twenty or thirty men or women steady-eyed, firm-lipped, even looking an inch or two taller and, every one endowed with all the energy necessary to inspire each of those patients with some of her own hope and to help on all her younger comrades; even to shedding a little of her enthusiasm into the somewhat callous dispositions of the scrubber and wardmaid! Now, how does it happen?

There is, of course, a certain weeding out process. It is not in every probationer, however eager, to develop a knack of managing people and things in the road it is desirable they should take. There may be, for instance, an incontrollable nervousness, which unfits one for taking command; or an ill-balanced temper in another, which can neither brook authority nor wield it with success. But with some few exceptions it will be found that at the end of three years in hospital the average girl will have become a capable, self-controlled woman, a nurse with plenty of "go" in her. Where does the change come in?

Someone says, "It is the training." Well, so it must be. Yet it is not the poring over books of elementary physiology, nor wielding the broom day by day, nor yet the bed-making, nor medicine-giving that does it altogether. But it is the steady routine, the enforced punctuality and order, the restraint of tongue and even of demeanour, the example of those further on in the course, which go a great way to make the competent nurse, the woman who can be trusted in an emergency to keep a steady head, and in the daily round to fulfil faithfully the day's duties.

Even more than the influence of the practical training in making the nurse is the sense of being face to face with the realities of life, mostly, it is true, with its tragedies. No girl can fail to be impressed with the sorrows of the world, as she sees the mother taken away from her little ones, or the man laid on a sick bed for months or years. Her sympathies are called out, and her patients unknowingly help to form her character on the best lines. They make demands on her, too, very often for advice, thus giving her the habit of looking at a thing on more than one side. Indeed, it is likely that the patients have more to do with the training of a nurse than the nurse quite realises. Their querulousness is met by her patience, their vacillation by her will, their weakness by her strength. And in time patience may even approach her "perfect work."

### To Nurses.

We invite contributions from any of our readers, and shall be glad to pay for "Notes on News from the Nursing World," "Incidents in a Nurse's Life," or for articles describing nursing experiences at home or abroad dealing with any nursing question from an original point of view, according to length. The minimum payment is 5s. Contributions on topical subjects are specially welcome. Notices of appointments, letters, entertainments, presentations, and deaths are not paid for, but we are always glad to receive them. All rejected manuscripts are returned in due course, and all payments for manuscripts used are made as early as possible after the beginning of each quarter.

### Central Midwives Board.

A meeting of the Board was held on Tuesday, April 10, and there were present Dr. Champneys in the chair, Dr. Dakin, Mr. Fordham, Miss R. Paget, Mr. Parker Young, and Miss Wilson.

Dr. Champneys was re-elected Chairman of the Board. It was decided to elect an Honorary Treasurer, and, on the motion of Miss Wilson, Mr. Fordham was asked to fill the post.

A letter from Mr. C. J. Wright, one of the Board's Examiners, as to additional fees for examiners in certain cases was referred to the Standing Committee.

A letter was received from the Hon. Secretary of the Irish Matrons' Association, and a similar letter from the Royal College of Physicians, Ireland, requesting the Board to hold examinations in Dublin at least three times a year. It was felt that as Ireland had expressly desired to be untouched by the Act, the Board could not justly incur any expenditure on its behalf, since, if any expenditure did fall on the Board in this respect, it would not be recoverable from Irish counties. It was decided not to administer the benefits of the Act outside the area to which the Act applies.

Sir William Sinclair had given notice to move the following resolution :—

"That a new rule in the following terms be inserted in Section C :

"'No one shall be permitted to begin the course of instruction, either theoretical or practical, until she has produced evidence satisfactory to the Central Midwives Board that she had received sufficient elementary education to enable her to read a text-book and take notes of cases. But this rule shall not apply to pupils who have received training in general nursing for at least one year.'"

In his unavoidable absence Dr. Dakin proposed and Mr. Fordham seconded that the motion be considered. The Chairman said that Sir William Sinclair's desire was to prevent women going through a course of training who were really too illiterate to profit from it. It was found that there was no absolute standard of efficiency obtainable through the Board of Education, and he (the Chairman) did not feel that the time was come when they could institute a sort of preliminary entrance examination, which had been, he thought, in the mind of Sir William. This was the general feeling of the Board. After some discussion Mr. Fordham moved that the old rule, that "Any candidate who during the examination shows a want of acquaintance with the ordinary subjects of elementary education may be rejected on that ground alone," should be retained, with a reference to Schedule Form V, and that, further, Schedule Form V, which is the form for a certificate of having attended a course of instruction, should set forth that the candidate "has shown that she possesses sufficient elementary education to enable her to read and take notes of cases." Dr. Dakin seconded the motion. The first part of the resolution was carried, but there was considerable divergence of opinion over Form V, some feeling that it was unfair to lay such responsibility on the lecturer, and that the real end desired—to prevent unsuitable women from taking up the work—would not be attained. The motion was eventually carried, the Chairman giving the casting vote.

Miss Wilson moved that Rule E 16 of the new rules, restricting the occasions upon which a midwife may lay out the dead, be altered so as to forbid her laying out "a body upon which a post-mortem examination has been made." She said that the police sometimes requested midwives to lay out the dead after such an examination, and that, being in the greater number of cases untrained women, they were not able to judge whether it would be dangerous to do so. This was carried unanimously. A letter from the London County Council was read, urging that it be provided that thorough disinfection take place after laying out of the dead. The following clause was therefore inserted : "After laying out a dead body she must undergo adequate cleansing and disinfection."

A long discussion took place on the wording of the rule relating to a midwife's sending for medical aid under certain conditions. The old wording : "A midwife must decline to attend alone, and must advise that a registered medical practitioner be sent for," had been the cause of much confusion, and frequently resulted in neglect of the patient. Eventually it was altered to read : "A midwife must explain that the case is one in which she must advise that a registered medical practitioner be sent for, and must hand to the husband, or the nearest relative or friend present, the form, properly filled up and signed by her, of sending for medical help, in order that this may be immediately forwarded to the medical practitioner. If for any reason the services of a registered medical practitioner be not available, the midwife, if the case be one of emergency, must remain with the patient and do her best for her until the registered medical practitioner arrives, or until the emergency is over. After having complied with the rule as to the summoning of medical assistance, the midwife will not incur any legal liability by remaining on duty and doing her best for her patient."

With respect to exemption of certain institutions from Section E, the rule was amended to read : "The rules or parts of rules in this section which are marked with an asterisk shall not apply to midwives exercising their calling under the supervision of a duly appointed officer within hospitals, workhouses, or Poor-law infirmaries, being institutions approved by the Central Midwives Board." The rules asterisked refer to the superintendence of the local supervising authority over midwives, notification of cases, and length of lying-in period. It was felt that the rule in its new wording would at least bring up the whole question before the Local Government Board in a concrete form.

A new rule was added empowering the Central Midwives Board to suspend a midwife from practice when a *prima facie* case of a serious character has been established by the local supervising authority, and to further enable the Board to suspend, after investigation of the case, where now they only censure.

### Appointments.

Barnstaple Infirmary.—Miss Catharine McLean has been appointed sister. She was trained at St. Thomas's Hospital, London, and has since been sister at Torbay Hospital.

Birkenhead Infirmary.—Miss J. S. Cockrell has been appointed lady superintendent. She was trained at St. Marylebone Infirmary. She has since been sister of the female medical and ophthalmic wards and sister of the female surgical and assistant matron and home sister in the same institution.

Borough Isolation, Huddersfield.—Miss Ethyle Cross has been appointed charge nurse. She was trained at the Manchester Children's Hospital, Pendlebury, and has since been staff nurse at the Clayton Vale Hospital, Newton Heath.

Bierley Hall Small-pox Hospital, Bradford.—Miss Jeannie Brooke has been appointed nurse matron. She was trained at the Sanatorium, St. Helens, and at the Sheffield

Royal Infirmary. She was afterwards sister at the City Hospital, Park Hill, Liverpool, and performed the duties of night superintendent. She has since been sister at the Bradford Eye and Ear Hospital.

Bury Infirmary.—Miss Linda Borth has been appointed night sister; Miss F. Freeman, sister of male and children's wards; and Miss C. Moore sister of the female ward. Miss Borth was trained at the Royal Infirmary, Preston, and has also received two years' fever training. She has since been senior staff nurse at the Southern Hospital, Manchester, and temporary sister at Highfield Infirmary, Liverpool. Miss Freeman was trained at Hull Royal Infirmary and Queen Charlotte's Hospital, London. She has since been staff nurse at Bromley Cottage Hospital, sister at the Victoria Hospital, Blackpool, and sister at Salisbury Infirmary. She holds the certificate of the Central Midwives Board. Miss Moore was trained at Burton-on-Trent General Infirmary, and has since been charge nurse at Carmarthenshire General Infirmary and sister at Stockton and Thornaby Hospital.

Cottage Hospital, Blandford.—Miss Rosa Newton has been appointed staff nurse. She was trained at the Salop Infirmary, Shrewsbury. She has since done private nursing in connection with the Birmingham and Midland Counties Institution for Trained Nurses; also at Worcester and at Stafford.

Cottage Hospital, Petersfield.—Miss Laughlin has been appointed matron. She was trained at the Royal Free Hospital, London, and has since been sister at Dulwich Infirmary and the Royal Victoria Hospital, Belfast; night sister at Chichester Infirmary; and nurse matron of the Cottage Hospital, Fordingbridge.

Fermanagh County Hospital.—Miss Josephine Porter has been appointed matron. She was trained at Leeds General Infirmary.

Highfield Infirmary, Liverpool.—Miss Janet C. Ball has been appointed assistant matron. She was trained at Westhulme Hospital, Oldham, and at the General Hospital, Leith. She has since been theatre sister at the Infirmary, Greenock, and sister and assistant matron at Lightburn Hospital, Shettleston, Glasgow.

Mansfield and Mansfield Woodhouse Hospital.—Miss A. Boss has been appointed nurse matron. She was trained at Ancoats Hospital, Manchester, and the Manchester Maternity Hospital. She has since been staff nurse at Ancoats Hospital, Manchester, charge nurse, and subsequently matron of the Hospital for Women, Derby.

Poplar and Stepney Sick Asylum.—Miss Edith Lizzie Rowe has been appointed junior assistant matron. She was trained at Southwark Infirmary, East Dulwich, and has been sister at Bethnal Green Infirmary, sister at Camberwell Infirmary, and night superintendent at St. Pancras Infirmary.

Royal Alexandra Infirmary, Paisley.—Miss I. Macdonald has been appointed sister. She was trained at the Royal Southern Hospital, Liverpool, and has since been sister at the Manchester Southern Hospital for Women and Children.

Stockton and Thornaby Hospital.—Miss M. E. Chesters and Miss L. Weir have been appointed sisters. Miss Chambers was trained at Sheffield Royal Infirmary and has since been sister at the Stanley Hospital, Liverpool, and sister at the Royal Eye Hospital, Manchester. Miss Weir was trained at University College Hospital, London, where she has since been staff nurse. She has also been night superintendent at the British Hospital, Cannes.

### Everybody's Opinion.

### NURSES' SOCIAL UNIONS.

Miss E. L. C. Eden writes: May I state that the "loan collection of nursing appliances," alluded to in your article last week, is not to lend to patients. It is a collection of up-to-date nursing requisites, foods, and pamphlets lent for exhibition at the meetings. Nurses can study the newest productions of the best firms and make a note of any address they require. I consider this an important feature of the Nurses' Social Union. Any further information about the Union can be obtained from Miss Josephs, Woodlands, Holford, Bridgwater.

### A QUESTION OF RESPONSIBILITY.

"A Troubled Nurse" writes: I am staff nurse in a fever hospital. The matron went away and I was left in charge. We have two blocks, one for scarlet fever, the other for diphtheria or typhoid, as the case may be. There are only two of us, a probationer and myself. Since the matron's absence we have been contented with one hour off-duty every other day. When the probationer was off I could not let the scarlet patients look after themselves; I was bound to come in contact with them. When I was off-duty the probationer looked after the diphtheria patients. A diphtheria patient contracted scarlet fever. Would any matron please tell me in next week's Hospital, am I or the sanitary authorities to blame for this?

### OPERATIONS ON MALE PATIENTS.

"F. R. B." writes: I was trained in a large general infirmary, and there was no medical school attached. I was theatre nurse for about four months. A patient was brought up for operation of amputation of penis and removal of glands. The sister of the ward came with the patient and remained till it was over, also the theatre sister and myself were there. I may say that another operation was in progress at the time, also another being got under. Sister and I were kept busy. As a district nurse I was asked to assist at an operation for removal of tumour from scrotum—patient laying on his back. I see no reason why a nurse should not be at hand to render whatever assistance is necessary. Of course the patient is always under an anæsthetic at the time.

### FEVER TRAINING IN GENERAL HOSPITALS.

"Another Trained Nurse" writes: I have often met well-trained nurses from London and provincial hospitals who have said how pleased they were to come to one of the Metropolitan Asylums Board Hospitals to learn ward administration as well as the fever work, and also to be able to thoroughly learn ear and nose syringing. Why did "Trained Nurse" comment on what the matron of Park Hospital had said? She was only answering the Commissioner's questions, not stating that the work generally was done better there than in many other hospitals.

### NIGHT NURSES' HOURS.

"Old Westminster" writes: If "A. B. C." had carefully read my letter she would see that I do not think twelve hours night duty too long. How I do wish the "nurses" who are so badly treated would leave the work for the many others who love it, as well as get their living by it? There would be fewer complaints then about the behaviour of nurses in public places, and in private houses, which, I think, would protect our uniform as much as anything. It is generally easy to judge between a steady-going trained nurse and a flighty, half-trained woman or a nursery woman, be she ever so nice. The public are as quick in judging as we are.

### A NEW WAY OF DUSTING WARDS.

"A General Hospital Nurse" writes: I have often thought that there was something wrong about the present way of dusting hospital wards, and it has lately occurred to me that it ought to be possible to utilise the vacuum apparatus for removing the dust which accumulates in such a marvellous way. Every hospital has its engine-room, and surely it would mean but a small expense to instal the necessary machinery. I am not a prophet, but I venture to predict that the nurses of the future will laugh at our present ways when they manipulate their vacuum dusters every morning.

### TRAINED NURSES AND UNTRAINED WOMEN.

"A St. Thomas's Nurse" writes: I regret that I was prevented writing last week in answer to the several sensible letters following mine on the subject of "Trained and Untrained Women." Some, I find, have lost the purport of my letter. I do not wish anyone to think that "nursery maids and generals" cannot be made nurses by training. I still maintain that it is a cruel thing for women untrained to be accepted at cases wearing our uniform. The matron of the "Fever Hospital" also appears to think that I abuse fever nurses generally; certainly not. I consider them noble women on the whole, but in the instance I mentioned they are untrained and simply go in for fever work because of the high salaries attached. As to the letter, "Good Form though in Uniform," whether written by a male or female, I consider it not worth comment. It is simply made up of quotations. I come to the conclusion that unless the London hospitals settle uniformity with trained nurses' uniform, nothing will prevent servants dressing as trained nurses.

### WAITRESSES ATTIRED AS NURSES.

"Matron of a Cottage Hospital" writes: It is with great indignation that I read a "Sister's" account of the way the professional nurses' uniform is being at present copied by the waitresses at the Bungalow Tea Rooms, Bournemouth. Surely there are enough suitable costumes obtainable without taking ours! This is the first time I have heard of our indoor uniform being imitated, except, of course, where nursing exhibits are shown, and I feel sure that nurses as a body will rise up against such a gross injustice to our profession. Why should not our uniform be protected by the State? It is enough to have to put up with seeing the numerous slovenly nurse-maids attired in our outdoor uniform parading the country walks and towns. I felt positively ashamed for my profession some few months ago to see the numbers of these nurse-maids scattered over the Clifton Downs, some of their number behaving certainly not as becomes the nursing profession.

### LINSEED-MEAL POULTICES.

"A. B." writes: I am much interested in what a matron and sister state in reference to linseed-meal poultices. I have had many years' experience in nursing, also in poultice-making, in which I am considered rather to excel. I always make my poultices by first warming the basin, then putting in my meal, adding water, and stirring at the same time, doing it very quickly. I have no trouble in getting them the right consistency to spread and without any waste or lumps. I can generally remove them without any lumps being left on the skin or crumbs in the bed. I always use a little oil, because I think that it is soothing to the skin, especially where the medical man requires long and frequent poulticing. I should like to know why this method is considered injurious. Some years ago I nursed for a very popular medical man a few miles from London. His method was to put into a saucepan just the quantity of boiling water, stir in the meal to the proper consistency, let it boil, then turn out on to a piece of flannel and spread till the flannel was completely covered. He made a point too of seeing that the nurse did it as he wished. My experience leads me to prefer his method, or my own, to the method we were taught, because I am quite sure that if I were making a large poultice for pneumonia or peritonitis by the time I had dredged in the meal there would not be much warmth in the poultice. I consider the method we were taught perfect for a small poultice. In giving instruction I always teach that way as correct, but in practice I always use my own. If it is really injurious, which I am not at all inclined to believe, I must have done a lot of harm rather than of good in my time!

### His First Easter Dawn.

Many called the grave, reserved night sister cold and hard; they were those who judged by appearance and did not take the trouble to get below the surface.

Certainly all the latent motherhood in the woman had been called out by the wee mite of a few months old lying quietly panting his little life away up in the children's ward. None seeing her as she bent softly over the cot night after night and gently moved the child, would have called her callous. He seemed to have some instinct that he comforted her sore heart as he held out his little arms to her, or feebly clutched at her finger. In spite of all the loving care he had she saw him fading, and just as the Easter Dawn was breaking she knelt alone by the bed while the nurse moved softly among the other cots of "Babyland," and in the quiet hush of night might almost be heard the rustle of angel wings coming to bear the baby "home again."

Suddenly the blue eyes opened wide and looked full into the brimming, weary ones watching him; the pained look passed and a radiant smile lit the little face as his spirit passed to the God who gave it. Gently the night sister bent and kissed the golden curls and two bright drops fell which only the angel saw.

The nurse passing by gazed tenderly on her little charge: "Sister," she said, "I wonder why he ever came into the world at all only to leave it so soon."

Perhaps the night sister might have replied "To soften my pain and to keep my heart from growing hard with the hurry and fret of life."

Later she carried the little white-robed form to the beautifully decorated chapel along the corridor, and there among the tall pure lilies and starry marguerites, she placed him for a little time on the altar step and kneeling above him laid her burden at the feet of the risen Christ.

The chancel was flooded in colours of crimson and purple and gold with the light of the rising sun, and surely there was rejoicing in heaven that the child had fulfilled his mission and returned to the God who sent him.

### Presentations.

Royal Cornwall Infirmary, Truro.—At the Royal Cornwall Infirmary, Truro, last week two presentations were made by the nursing staff. The first was to Miss Davies, matron, who was presented with a silver-mounted manicure set, glove-stretcher and hook, button-hook, and shoe-horn. The other presentation was to Miss E. A. Pidgeon, sister of the male wards and theatre, to whom the matron and staff gave a silver-mounted Swan "Stylographic" pen.

### Death in our Ranks.

We regret to hear of the death, on April 7, of Miss Margaret Kendal, sister in Queen Alexandra's Imperial Military Nursing Service. Miss Kendal, who was only twenty-nine years of age, was engaged in nursing at the Royal Military Hospital, Wynberg, South Africa.

### Wants and Workers.

District Nurse, Cawston Lodge, Cawston, Norwich, would be most grateful if some kindly disposed lady would give or sell cheaply a truss for a very poor deserving man of slight build who cannot afford to buy and yet suffers greatly from rupture right groin.

### A Book and its Story.

### A SILENT HEROINE.*

Mrs. Fred Reynolds' last book is in every way charming, and an advance on any previous one of hers that has come before us. It is written with refinement and insight, showing real sympathy with Nature in its varying moods, and no less fellow feeling for suffering humanity, as is seen in the delicate delineation of Psyche, the deaf and dumb heroine of the aptly named novel, "In Silence."

At once we are put in touch with the moorland scene and the pretty, pathetic figure of Psyche: "A silent world and the child, herself no less silent . . . a world of heather, heather softly purple, blushingly pink, wanly grey. Acres and acres of it, broken here and there by tracks of bracken, bronzing already under the scorching August sun that shone from a sky of hot, melting blue." Standing among the rocks that edge the little moorland stream, intent on the capture of pale-winged moth, she is lost to the approach of a stranger who is coming towards her and stops in admiration at the vision of childish liveliness. "A tall, well-grown youth, half man, half boy, stopped for a moment, a kindly admiration showing in his honest brown eyes. The child was indeed worth looking at. She had risen now to her feet, and stood, half turned away, lightly poised, as though herself in the act of flight, intent on the moth now raised on a rosy finger-tip against the blue of the sky. Her gown, a faded, pinkish blue, fell in soft folds about her lithe figure; . . . her hair, the brown of ripe wheat stalks, clustered in close curls about her small head; the sun had bleached the curl-tips into palest gold; her lashes curled long and dusky against the clear sky. 'A lovely child,' said the youth to himself. Then aloud: 'Am I right for the lake, little lassie?' . . . The child, however, paid not the slightest heed to the question. The boy repeated it. Still no reply. The moth, at length spreading its delicate wings for flight, was evidently of far greater interest than he. Slightly piqued the questioner passed on, but not before he had assured himself that the eyes under the dark lashes were blue. And what a blue! 'Like liquid sapphires,' said the boy to himself as he went crushing down through the heather and rushes. The long trout he bore, bending to his movements, flashed back the noonday sun."

Adrian Harlech makes his way to the margin of the lake and finds that his boat which he had left moored there had disappeared. He mounts a rock and discovers that she has slipped her moorings and is drifting across the lake. Preparing to swim across in pursuit he sees Psyche coming down the lake in a little boat. His efforts to arrest her attention by shouts being useless he attempts signs, which are effectual in making her understand that he has lost his boat. She turns back, rows up to it, attaches her tiny craft to the stern, ships her own oars, and taking the larger ones puts the boat about and makes for the shore. "By jove!" said the watcher, "what a picture it would make." "(A picture of childhood and all joyousness was in his thoughts; he had not as yet fathomed the tragedy so often lurking behind the joy of life.)" Following Psyche, after she had landed and the boats were safely grounded, they come to the farmhouse where Psyche is living in charge of an old servant of her mother's family. Adrian has not yet fathomed the mystery of the child's silence. Her look of unusual intelligence did not suggest any deprivation of the sense of hearing. But "could she be dumb?" As he sits in the house room of the farmhouse, tended by the hospitable mistress, the thought casts a sadness on the surroundings, which is ably presented in the following passage: "The tall corner clock ticked a sombre undertone, a little heap of peat smouldered dully on the hearth, the broad triangular splash of sunshine that fell on the flags through the open door only served to render the remainder of the room more dim. Strange forms seemed to lurk in the shadows, holding he knew not what presage of ill or sorrow. All was so still, only a bee droned drowsily against the window-pane. . . . A silent house in a world of silence."

Adrian learns the history of Psyche from his hostess. She is an orphan. Her mother had died some years before; her father did not live to see his little daughter. When it was found that Psyche's irresponsiveness arose, not from inattention, which it was at first put down to, but from congenital deafness, she is taken, at the advice of a famous children's doctor, to the farmhouse on the fells to be under the tender care of "Mother Thwaite." Her guardian and uncle, Sir George Raiburn, is devoted to his little niece, and when, in the gloom of the house following the death of her mother, the child had visibly drooped, he had felt "that unless something were done, the child would slip away into the shadows after that dear lady her mother. . . . She wanted the sunshine—that only. They had taken it from her, and she had no power to voice her woe. So she sat, a little huddled heap, in the midst of the wide floor, with limp curls and dulled blue eyes, and no one, meant they ever so well, could approach the barred fortress of her soul. . . . So it came about Sir George bethought him of the faithful nurse of his own sons, and the little Psyche left behind her the wide gloomy house and the sombre-robed women, and also, by the most thoughtful of great men's very special order, her own black frocks, and was conveyed by Sir George in person to the upland valley, and straight to the heart of Mother Thwaite herself." The spiritual isolation imposed by deafness, and its compensations, are very fully realised and admirably expressed by the author. "It has been said that each one of us in reality dwells in a world apart. The language we have made for ourselves, to escape each somewhat of his soul's loneliness, does but draw us a trifle nearer. Though two speak alike of light and darkness, the darkness of the one may not in reality be that of the other; and the light of the one the other may have no conception of. If all then are held apart by the dense fog that wraps each little entity unto itself, for Psyche there were not even hands held out in the darkness; for her there was not even the speech which most of us misuse, but to conceal our thoughts. . . . And if by reason of her affliction the child was denied much of the joy of life, by the same reason she was spared much of its sadness. No harsh voices ever penetrated into her life, no nursery scolding sent her with bleeding heart and long-drawn sobs to a tear-stained pillow. Though hers was a silent world it was also one of undisturbed peacefulness. If there was for her no Heaven, there was likewise no Hell." The very touching story of Psyche will be read with interest by everyone who is interested in the study of lip language. The training she receives at a special school, after four years, enables her to speak, and understand without hearing what others are saying. Her unusual intelligence helps her to attain a fuller realisation of the benefits bestowed by the training she has received, and to long to establish a Home of her own for the education of children who are deaf and dumb. We cordially recommend Mrs. Reynolds' sweet story.

---

* "In Silence." By Mrs. F. Reynolds. (Hurst and Blackett. 6s.)

### Notes and Queries.

**REGULATIONS.**

**The Editor is always willing to answer in this column, without any fee, all reasonable questions, as soon as possible.**

**But the following rules must be carefully observed.**

1. **Every communication must be accompanied by the name and address of the writer.**
2. **The question must always bear upon nursing, directly or indirectly.**

**If an answer is required by letter a fee of half-a-crown must be enclosed with the note containing the inquiry.**

*Paint for Hospital Walls.*

(32) A correspondent suggests that the matron who asked about paint for hospital walls may be glad to hear of "Velure." It is a very smooth, uncrackable, elastic paint. It is sold by Chancellor and Company, 13 Clerkenwell Road, E.C.

*School for Midwives.*

(33) Is there a training school for midwives at West Kensington, London, or is it best to train at Queen Charlotte's? Would it make any difference afterwards where one was trained, so long as one passed the Central Midwives Board.—*U. M. P.*

The certificate of the Central Midwives Board is in itself a guarantee of efficiency, but there are numerous institutions approved as training schools by the Board, of which a list can be obtained on application to the Scientific Press, 29 Southampton Street, Strand, W.C.

*Registration of Midwives, etc.*

(34) I am a trained midwife and monthly nurse, but, having been out of touch some time with nursing matters, I should be glad if you will tell me where I can obtain full information with respect to "Registration of Midwives," "Central Midwives Board," and "Holt-Ockley" rules, and any other matters likely to be useful.—*R. A. D.*

You would obtain all information from the offices of the Central Midwives Board, 6 Suffolk Street, Pall Mall, and for information concerning the Holt-Ockley system, which is now merged into the Cottage Benefit Nursing Association, write to the Secretary, Denison House, Vauxhall Bridge Road, S.W.

*Nursing in Rome.*

(35) I wish to do private nursing in Rome. Can you give me information in regard to the institutions where certificated nurses are employed?—*F. J. E.*

St. Paul's Home, Via Palestro, and the Anglo-American Nursing Home, 265 Via Nomentana, both employ English nurses.

*Feeble-minded Girl.*

(36) Can you tell of a home where a feeble-minded girl of 16 can be admitted? Although mentally deficient, she can do house work, and her parents, who are of the working class, would be willing to pay 5s. weekly for her.—*E. M. A.*

Write to the Secretary, National Association for Promoting the Welfare of the Feeble-minded, 53 Victoria Street, S.W., and to the National Training Home for the Feeble-minded, 36 King William Street, E.C.

*The Nurses' Union.*

(37) Can you give me the address of the Nurses 'Union?

The London secretaries are Miss Greer, Grove House, Regent's Park, N.W., and Miss M. Dashwood, 5 Cambridge Gate, Regent's Park, N.W., either of whom will gladly send you all particulars.

*Children's Recreation Home.*

(38) Is it correct that Parkwood, Henley-on-Thames, where many nurses were received up to the end of March, is now available for children?

Yes, from the beginning of April to the end of October Mrs. Henry receives at Parkwood a limited number of children of both sexes between nine and twelve, with the object of affording them a fortnight's holiday in the country. Her object is to help parents who find it a difficult matter to educate their children, and whose means will not allow them the extra expense of a holiday. All applications for full particulars should be addressed to the matron.

**Handbooks for Nurses.**

| | Post Free. |
|---|---|
| "How to Become a Nurse: How and Where to Train." | 2s. 4d. |
| "Nursing: its Theory and Practice." (Lewis.) ... | 3s. 6d. |
| "Nurses' Pronouncing Dictionary of Medical Terms." | 2s. 6d. |
| "Complete Handbook of Midwifery." (Watson.) ... | 6s. 4d. |
| "Preparation for Operation in Private Houses." ... | 0s. 6d. |

**Of all booksellers or of The Scientific Press, Limited, 28 & 29 Southampton Street, Strand, London, W.C.**

### For Reading to the Sick.

"AFTERWARD."

And now we fight the battle,
But then shall wear the crown
Of full and everlasting
And passionless renown:
And now we watch and struggle,
And now we live in hope,
And Sion in her anguish
With Babylon must cope.
But He Whom now we trust in
Shall then be seen and known,
And they that know and see Him
Shall have Him for their own.

*Bernard of Cluny.*

There is a wondrous power of explanation in "afterward." Things do not seem to us to-day as they will seem to-morrow. This is the key which the Scriptures give us for the solution of the strange mystery of affliction. "No chastening for the present seemeth to be joyous, but grievous; nevertheless, afterward it yieldeth the peaceable fruit of righteousness." There are many things in God's way with his people which, at the time, are dark and obscure, but which the future makes clear and plain. To-day's heavy clouds to-morrow are gone; and under the bright shining of the sun, and the deep blue of the sky, the flowers are sweeter, the grass is greener, and all life is more beautiful. To-day's tears to-morrow are turned to lenses through which eyes, dim no longer, see far into the clear heavens, and behold the kindliness and radiance of God's face.

One reason for the present obscurity of life is our ignorance, our limited knowledge. We know now only in part: we see only in a mirror darkly. We have learned merely the rudiments, and cannot understand the more advanced and abstruse things. . . When we stand, at length, at the end of our school-days, the old, confusing pages will be plain and clear to us, as childhood's earliest lessons, though hard at the time, are afterward to ripe, manly wisdom. Then we shall see that every perplexed line held a golden lesson of wisdom for our hearts.

We are all like little children. God writes in poetry which, no doubt, is very beautiful, as his eyes look upon it, and read its sentences; but we must wait to learn more before we can read the precious truths and golden thoughts which lie in the lines. In our sorrows and disappointments good men come to us, and tell us that the Lord doeth all things well; that there is some blessing for us in every bitter cup; that the strange answers we get to our prayers are the very best things of God's love, though so disguised. We open the Bible, and we find there the same assurances; but we cannot see the blessing, the good, the love, in the painful and perplexing experiences of our lives. To our dim eyes all is darkness, and our faith is well-nigh staggered. Then our Lord's word comes to us, "What I do, thou knowest not now; but thou shalt know hereafter." "Afterward" is the key. Possibly in this world, certainly in the great "hereafter" of heaven, we shall see that every providence of God, even the providences that were painful, and that seemed adverse, meant blessing and good.

*Dr. J. R. Miller.*

## Figures and Tables

**Fig. 12. f1:**
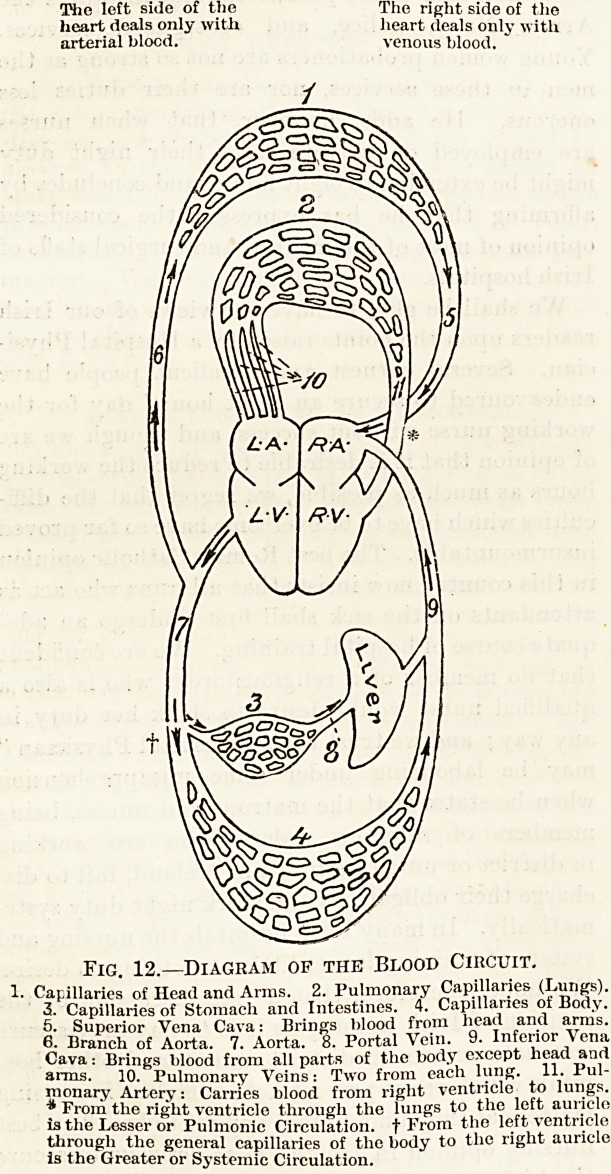


**Fig. 13. f2:**
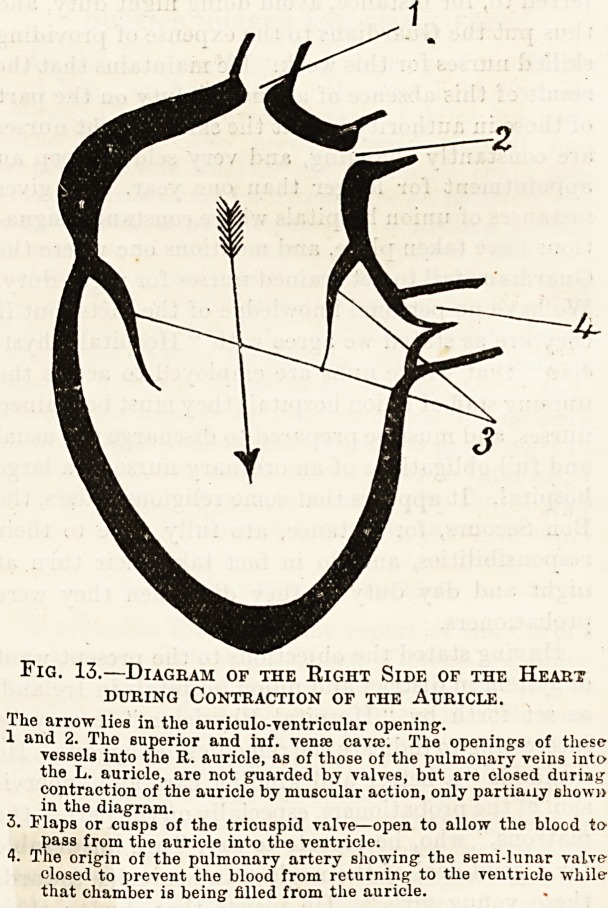


**Fig. 14. f3:**